# Dermatological Conference

**Published:** 1871-05

**Authors:** 


Dermatological Conference.—A dermatological conference, to be held, say, in London, would be an event of much interest. Great Britain and Ireland, Germany, France, Italy, America, etc., ought to be well represented. Many important matters could engage the attention of the conference, as, for instance, a classification of skin diseases, the settlement of the vegetable parasitic question, the necessity for the licensing corporations recognizing dermatology, etc., etc. We merely mention the above idea; if thought feasible, we hope that steps may be taken to act on the suggestion.—*Journal of Cutaneous Medicine.*

